# Post-tuberculosis (TB) Unilateral Left Lung Damage: TB Misdiagnosis and Mismanagement

**DOI:** 10.7759/cureus.95832

**Published:** 2025-10-31

**Authors:** Katanekwa Njekwa, Nsala Sanjase, Ethel Kamuti, Kondwelani Mateyo, Monde Muyoyeta

**Affiliations:** 1 Clinical Trials Unit, Center for Infectious Disease Research in Zambia (CIDRZ), Lusaka, ZMB; 2 TB Programs Department, Center for Infectious Disease Research in Zambia (CIDRZ), Lusaka, ZMB; 3 Internal Medicine Department, University Teaching Hospital of Zambia, Lusaka, ZMB

**Keywords:** post tuberculosis lung damage (ptld), pulmonary mycetoma, radiologic findings, spirometry, tuberculosis (tb)

## Abstract

The extent of lung damage post-tuberculosis (TB) infection varies greatly, resulting in impairment of pulmonary structure and/or function. We present a case of TB misdiagnosis and mismanagement in a 53-year-old black African male living with HIV on anti-retroviral therapy (ART) with post-TB unilateral left lung damage. He presented with a one-month history of intermittent symptoms of fever, night sweats, right-sided chest pain, exertion intolerance, and hemoptysis, and was commenced on anti-TB treatment (ATT) at a local clinic despite the two negative TB infection laboratory results. Further evaluation, later by TB specialists, showed an underweight male in no obvious respiratory distress (at the time of examination), normal vitals, positive Trail’s sign, chest asymmetry, and a displaced apex beat. Radiological findings included a collapsed left upper lung with extensive fibrosis and mediastinal shift on chest X-ray. Chest CT scan findings revealed bronchiectasis, left lung collapse with left upper lobe cavitary lesion containing an irregular mass, shift of the mediastinum to the left, and compensatory right lung hyperinflation. Laboratory findings showed a normocytic anemia. *Neisseria* species was cultured in sputum. GeneXpert on sputum was negative (twice) for TB on this current evaluation. SARS-CoV-2 PCR was also negative. A pulmonary function test (PFT) using spirometry showed restrictive lung damage with forced vital capacity (FVC) (2.26 (47%)), forced expiratory volume (FEV_1_) (2.01(50%), and FEV_1_/FVC (88.93%) ratio. A diagnosis of post-TB lung disease (PTLD) was confirmed. The ATT was stopped, and the PTLD findings were communicated back to the staff at the TB/chest clinic where the patient was treated for TB. Based on the current laboratory results and symptoms, the patient was treated with fluconazole 200 mg orally once daily for two months, analgesics, mucolytics, azithromycin 500 mg orally once daily for three days, and supportive therapy. The patient was also reviewed routinely at the pulmonology clinic.

## Introduction

There is a call by the global community for a holistic, integrated approach in the management of tuberculosis (TB), including TB-associated impairment and disability [1]. Pulmonary TB (PTB) has been listed among the leading causes of respiratory morbidity [2]. The extension of TB treatment and support beyond cure is important to address any sequelae associated with TB [3]. Post-TB lung disease (PTLD) may result from cicatrization, alteration of parenchyma, bronchiectasis, and scarring of the lung, leading to respiratory compromise, which is a culmination of the complex interaction between the TB causative organism, the host, and environmental factors [4]. Some factors that have been observed to drive PTLD include social factors, immunocompromise, and inadequate effective prevention of TB infection and disease [5]. Unfortunately, the current global and regional report estimates of PTLD undermine the full extent of the problem [6]. In this report, we present a clinical case of TB misdiagnosis and mismanagement in the form of PTLD. In this case, the healthcare workers at the local clinic still continued to commence the patient on anti-TB treatment (ATT) based on his current symptoms suggestive of TB despite receiving two negative TB GeneXpert results. This is in addition to the positive two-time history of being treated for TB. Fortunately, during the patient's routine clinical reviews, during which further evaluation of his current TB treatment was done, it was discovered that the patient was incorrectly diagnosed as a case of TB. The patient was then linked to the TB disease specialists and further managed at the pulmonology clinic.

## Case presentation

In 2023, a 53-year-old African black Zambian male presented to the hospital with a one-month history of right-sided chest pain and a productive cough. The pain was described as dull, localized mostly to the right, and increased with excessive coughing. Coughing was observed mostly at night when he lay on his left side, and he had difficulty breathing more when he lay on his right side. The sputum was occasionally blood-stained and non-foul-smelling. The patient was also admitted to experiencing exertion intolerance and having fevers, night sweats, and dizziness. He also added that he had lost weight, which he described as a reduction in his body size, but denied loss of appetite, vomiting, and diarrhea.

He was a person living with HIV (PLHIV) and on combined anti-retroviral therapy (cART) (tenofovir disoproxil fumarate, lamivudine, and dolutegravir orally once per day) since 2021. He was first diagnosed with PTB in his sputum in 2007, and in 2020, a second time presumptively, on Chest X-ray, for which he reports having received ATT in both cases. He also gave a history of undergoing surgery (laparotomy) in 2017. There was no history of COVID-19, diabetes mellitus, or hypertension. He also gave a history of being vaccinated with the mRNA (Moderna) COVID-19 vaccine in a clinical trial and received three doses of the vaccine at month 0, 1, and a booster at month 6. He was an ex-smoker (10 pack-year history) and denied alcohol use. He was married and worked as a car mechanic. He resided in the densely populated peri-urban area of Zambia's capital city, Lusaka. 

**Figure 1:**
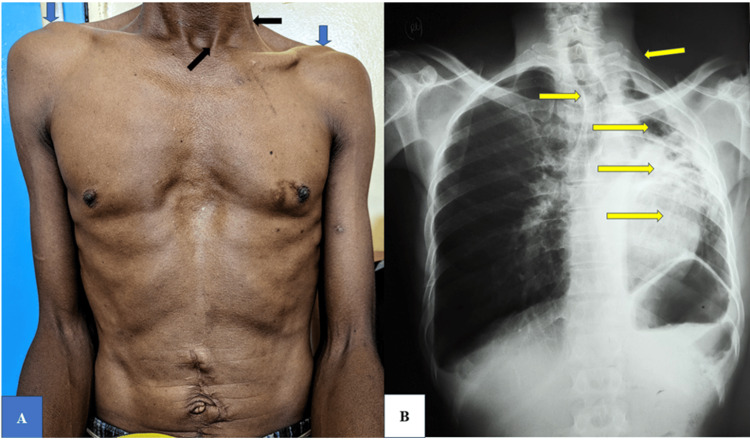
(A) Chest asymmetry observed, evidenced by the left lower acromioclavicular joint compared to the right (blue arrows). There is a positive Trail’s sign - prominence of the clavicular head of the sternocleidomastoid muscle on the left side (black arrow). Old midline surgical scar. (B) Plain radiological chest X-ray (analogue) (yellow arrows from up going downwards). Increased shadow prominence of the clavicular part of sternocleidomastoid muscle. Tracheal deviation to the left. Collapsed left upper lung. Fibrotic lung tissue. Suspected shifted mediastinum. Clear costophrenic and cardiophrenic angles. Clear right lung fields with some perihilar infiltrates.

Two GeneXpert tests conducted during this current episode were negative. His absolute CD4 count was 576/uL, and his viral load was undetected. A chest X-ray was ordered (Figure 1), and a diagnosis of TB was made. He was initiated on first-line ATT (fixed-dose combination of isoniazid (H), rifampicin (R), pyrazinamide (Z), and ethambutol (E)). Treatment was discontinued after two weeks, following guidance from TB specialists after presenting the patient's clinical/medical history and radiological findings, who suggested it to be a case of PTLD.

On general examination, he had a temperature of 36.7ºC, blood pressure of 102/70 mmHg, pulse rate of 96 beats per minute, and respiratory rate of 21 breaths per minute (no obvious respiratory distress). He appeared wasted with a BMI of 16.9 kg/m^2^. Respiratory examination revealed chest wall asymmetry (Figure 1A) and associated reduced air entry on the left side on auscultation. The cardiovascular examination revealed an inferiorly displaced apex beat in the sixth intercostal space with normal heart sounds. Abdominal examination revealed an old midline surgical scar, with no abdominal distension or abnormal palpable abdominal masses. The rest of the systemic examination showed no significant findings.

The laboratory examination of sputum showed it to be mucoid, with a Bartlett's score of +1. The sputum culture identified *Neisseria* species other than meningitidis or gonorrhoeae species, sensitive to chloramphenicol, ceftriaxone, ciprofloxacin, and azithromycin, and resistant to trimethoprim-sulfamethoxazole and meropenem. Antimicrobial susceptibility testing was performed using the Kirby-Bauer disk diffusion method, and the guideline that were followed to interpret results was the Clinical and Laboratory Standards Institute (CLSI) M100 standard. The manual conventional method involved the use of biochemicals, namely, (1) colony morphology on chocolate agar and blood agar, (2) Gram stain, (3) oxidase test, and (4) catalase test. With this method and its limitations, the laboratory was only able to rule out two species, which are meningitidis and gonorrhoeae, but was also unable to provide other specifics about the *Neisseria* species. 

Fungal infection was ruled out by culturing the specimen on a fungal medium called Sabouraud Dextrose Agar and Gram staining. The Cartridge-Based Nucleic Acid Amplification Test (CBNAAT) and Ziehl-Neelsen (ZN) stain were negative. Non-tuberculous mycobacteria were also ruled out using mycobacterial culture. The COVID-19 polymerase chain reaction (PCR) test was negative. The full blood count (FBC) showed a normocytic normochromic anemia (hemoglobin (Hb) 12.4 g/dL (13.7-17.7), mean corpuscular volume (MCV) 98.9 fL (77.6-98.1), and mean corpuscular hemoglobin (MCH) 33.5 Pg (23.1-33.2). The other FBC parameters, renal and liver function tests, were normal. 

**Figure 2:**
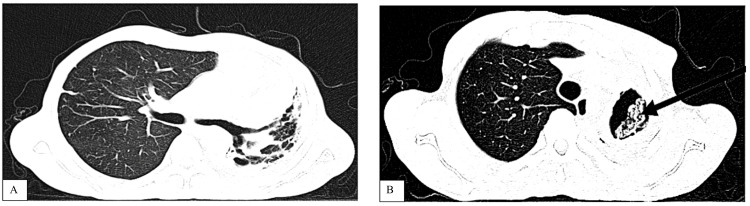
Chest CT-scan with the radiologist’s report. (A) Extensive left lung fibrotic changes with near total lung collapse and shift of the mediastinum to the left. Associated left lung bronchial wall thickening with dilatation. Left upper lobe cavitary lesion containing an irregular mass. Compensatory right lung hyperinflation with no obvious lesions. Trachea is slightly shifted to the left. No lymphadenopathy. Heart is not enlarged. No bone lesions. (B) Post-pulmonary tuberculosis changes showing bronchiectasis, left lung collapse, and left upper lobe cavitary lesion containing an irregular mass. Differentials for the irregular mass lesion within the cavity include necrotic lung tissue versus a mycetoma.

The radiological investigations, which included chest X-ray and computed tomography (CT) scan, were done (Figure 1 and Figure 2, respectively). A diagnosis of post-TB unilateral left lung damage with suspected infectious exacerbation was made. Spirometry test readings showed a forced vital capacity (FVC) (2.26 (47%)), forced expiratory volume (FEV_1_) (2.01(50%), and FEV_1_/FVC (88.93%) ratio consistent with restrictive lung disease. He was treated with fluconazole 200 mg orally once daily for two months, oral analgesics (unknown), mucolytics, and azithromycin 500 mg orally once daily for three days. Chest physiotherapy and routine reviews in the pulmonology clinic.

## Discussion

This case report is a classic case of TB misdiagnosis and mismanagement due to limited knowledge on post-TB sequelae or residual disease. The patient presented with long-standing symptoms consistent with those of active TB, and despite the negative GeneXpert sputum result, presumptive TB treatment was initiated based on the chest X-ray findings and symptoms during the first presentation to the local healthcare facility. The GeneXpert MTB RIF (Cepheid) ultra-platform was used to test for TB on both occasions in this case, which has improved sensitivity but lower specificity in patients with previous TB or in patients from high-incidence countries [7]. The lack of clarity in respiratory symptoms and radiographic abnormalities in patients previously treated for TB has been reported to result in substantial overtreatment in these patients [8]. In this case, the patient reported a history of being treated for PTB twice, of which his second diagnosis was made using a chest X-ray. Unfortunately, the full extent and impact of post-TB sequelae still remain uncharacterized in developing countries such as Zambia, where TB is endemic.

TB may result in impairment of respiratory function, which may present as chronic lung disease, especially in endemic countries [9]. Access to improved TB screening, diagnosis, and patient care still poses a major challenge in most parts of Zambia despite the ongoing strategies to improve patient-centered engagement [10]. The current model of TB patient care, defined by discharge post-treatment completion with no rehabilitation, healthcare advice, or routine review, may not be enough [11]. This has led to PTLD patients being missed by the healthcare systems, misdiagnosed, or undertreated. This is in the face of the success of the TB control and elimination program, anchored on the TB knowledge and attitude of healthcare workers [12]. The patient also gave a history of smoking (10 pack/year). Tobacco exposure has been reported to increase the risk of contracting TB, recurrence of TB, and impair the response to treatment of the disease, but may be confounded by socioeconomic factors [13].

The damage to pulmonary parenchyma structures post-TB disease may lead to frequent respiratory tract infections such as chronic pulmonary aspergillosis (CPA) and non-tuberculous mycobacterial (NTM) infections [14-16]. An assessment of the sputum using Bartlett’s grading system was found to be +1, indicating the presence of active inflammation [17-18]. In this patient, *Neisseria* species was isolated, which was sensitive to chloramphenicol, ceftriaxone, ciprofloxacin, and azithromycin but was resistant to trimethoprim-sulfamethoxazole and meropenem. Trimethoprim-sulfamethoxazole (cotrimoxazole) is routinely used as prophylaxis for *Pneumocystis* pneumonia (PCP) in PLHIV. In view of the hemoptysis, chest pain, and some of the constitutive symptoms the patient presented with, clinicians in their assessment of recurrent hemoptysis must consider other etiological factors in the processes of confirming or excluding the diagnosis of TB [14]. The FBC/differential count (FBC/DC) results reviewed a normocytic normochromic anemia with a reactive thrombocytosis (Table 1). The patient in this case report was treated with anti-fungal, analgesia, antibiotics, lifestyle changes (e.g., avoid triggering factors - heavy work, smoking, etc.), chest physiotherapy, and scheduled for routine reviews in the pulmonology clinic. Post-TB treatment discharge should include assessment of post-TB sequelae for earlier diagnosis and timely intervention. The TB damage to the lungs predisposes patients to not only TB relapse but also other complications, such as superimposed infections, COPD, or bronchiectasis, which may affect the patient’s quality of life [2,19-20].

## Conclusions

This case highlights a lack of appreciation for PTLD in the healthcare system, leading to inappropriate exposure to ATT treatment. One of the important factors contributing to the development of multidrug-resistant TB (MDR-TB). Unfortunately, this threat will persist for as long as structures are not put in place to identify, follow up, and manage PTLD.

There is a need for high-burden TB countries to develop post-TB treatment care strategies. Awareness of the burden of PTLD amongst healthcare providers is crucial for earlier diagnosis and prevention of misdiagnosis as TB relapse. In addition, strengthening easy access and linkage to timely consultations with TB and pulmonology specialists for case management of TB and other respiratory diseases could ensure accurate diagnosis, improved outcomes, and reduced ongoing morbidity.
